# Sublingual Immunotherapy with a Five-Grass Pollen Tablet in Adult Patients with Allergic Rhinitis: An Open, Prospective, Noninterventional, Multicenter Study

**DOI:** 10.1155/2015/584291

**Published:** 2015-08-16

**Authors:** Oliver Pfaar, Heinz-Gerd Richter, Ludger Klimek, Jochen Sieber, Meike Hadler, Efstrathios Karagiannis

**Affiliations:** ^1^Center for Rhinology and Allergology, 65183 Wiesbaden, Germany; ^2^Department of Otorhinolaryngology, Head and Neck Surgery, Universitätsmedizin Mannheim, Medical Faculty Mannheim, Heidelberg University, 68167 Mannheim, Germany; ^3^Private Office, 45130 Essen, Germany; ^4^Stallergenes GmbH, 47475 Kamp-Lintfort, Germany

## Abstract

*Background*. Although the safety and efficacy of sublingual immunotherapy (SLIT) with a five-grass pollen tablet have been demonstrated in randomized clinical trials (RCTs), these outcomes must always be evaluated in real-life medical practice. *Methods*. In a prospective, open-label, noninterventional, “real-life” study in Germany, we evaluated the safety, tolerability, and effectiveness of SLIT with a five-grass pollen tablet in adults with grass-pollen-induced allergic rhinoconjunctivitis. *Results*. 808 adults were enrolled between September 2008 and December 2009. 35.3% of the participants experienced at least one adverse drug reaction (ADR), the most common of which were mild-to-moderate gastrointestinal and respiratory disorders. Serious ADRs considered causally related to SLIT treatment occurred in four patients. Overall, the five-grass pollen tablet was considered to have good or very good tolerability by most investigators and patients. Treatment was associated with the relief of nasal, ocular, and bronchial symptoms and decreased symptomatic medication use. However, interpretation of clinical improvements was limited by lower atmospheric grass pollen levels during the study season (relative to the preceding season). *Conclusions*. In a large population of patients treated in real-life medical practice, SLIT with a five-grass pollen tablet was safe and well tolerated. The patient-reported symptom relief suggests that SLIT was associated with clinical benefits.

## 1. Background

Allergic rhinoconjunctivitis (ARC) is a major health issue worldwide and affects more than 500 million people in their daily lives [1]. Furthermore, allergic rhinitis is associated with an increased risk of developing secondary allergic diseases (such as asthma) [1]. Grass, weed, and tree pollens are responsible for the most common pollen allergies [1]. In principle, there are three treatment options for immunoglobulin E- (IgE-) mediated ARC: allergen avoidance, symptomatic medications, and allergen immunotherapy (AIT) [2]. However, AIT is the only therapeutic option capable of changing the natural course of allergic disease and has demonstrated long-term, disease-modifying effects [1, 2].

Traditionally, AIT has been administered via the subcutaneous route; this involves the inconvenience of frequent injections and is associated with a low but nonnegligible risk of potentially severe, systemic anaphylactic reactions [3]. Over the last few decades, alternative administration routes (such as the sublingual route) have been developed with a view to limiting discomfort and mitigating risk [4]. There is now substantial and growing use of sublingual immunotherapy (SLIT) in Europe and the USA [1, 2, 5]. This increase can probably be explained by SLIT's favorable safety profile and ease of administration [5, 6]. Indeed, SLIT has become a standard treatment for aeroallergen-induced ARC and is widely acknowledged to be efficacious, safe, and well tolerated [1, 6, 7].

The safety and efficacy of SLIT with a five-grass pollen tablet have already been demonstrated in adult and pediatric populations in large, double-blind, and placebo-controlled (DBPC) randomized clinical trials (RCTs) [8–14].

Although DBPC RCTs are considered to provide the best available clinical evidence, their patient inclusion and exclusion criteria are often highly restrictive and the number of recruited patients is often limited; hence, a trial population will tend to differ from the population of patients encountered in real-life medical practice [15]. Consequently, “real-life” studies are needed to assess “real-life” outcomes (which may conceivably differ from those observed in RCTs) [2, 16]. As a complement to RCTs, large-scale, noninterventional studies with patients treated according to an authorized medicine's summary of product characteristics (SmPC) are clearly of value when interpreting tolerability and effectiveness under real-life conditions [17]. The results of recent observational, noninterventional studies indicated that the effectiveness of SLIT formulations in daily medical practice life correlates well with the efficacy seen in randomized, controlled trials [18]. Recently, pooled safety data from adults, adolescents and children in the five-grass pollen tablet clinical development program, post-approval studies (including the noninterventional study described hereafter) and more than 6 years of real-life experience have been published [19]. Here we report on the adult postmarketing data in detail.

Following the marketing authorization of the five-grass pollen tablet in Germany in June 2008 and with a view to supplementing the results of RCTs, we performed a prospective, noninterventional study of the safety, tolerability, and effectiveness of pre- and coseasonal treatment in a large population of adult patients with ARC.

## 2. Methods

### 2.1. Study Design

This was a nonrandomized, prospective, open-label, multicenter, noninterventional study designed to assess the safety, tolerability, and effectiveness of pre- and coseasonal SLIT with a five-grass pollen tablet in real-life medical practice. The study was performed between September 2008 and December 2009 at 354 study centers in Germany. To ensure that the sample was representative, investigators were recruited from throughout the country. Patients were only included in the study if a decision to initiate SLIT with the five-grass pollen tablet had already been made. Consecutive cases were enrolled.

According to the German Drug Law [20], noninterventional studies conducted after the marketing authorization of a medicinal product have to be notified to the competent authority but are not subject of approval by an ethics committee or an institutional review board if the data collection does not exceed routine medical documentation. The competent authority for allergen extracts in Germany is the Paul-Ehrlich-Institute (PEI) which was notified in September 2008. Only data incidental to routine use of the five-grass pollen tablet in real-life medical practice were documented. Collected data were transmitted for analysis without disclosure of patient names or initials in a pseudonymized way. Thus, no data which would allow direct identification of a patient were available to personnel outside the investigators sites.

### 2.2. Patients

In accordance with the authorized indication [21], adult patients aged 18 and over with IgE-mediated, grass pollen-induced allergic rhinitis (with or without conjunctivitis or asthma), confirmed by a positive skin prick test and/or a positive serum titer of specific IgE to grass pollen, and clinically relevant symptoms were included in the study.

Patients were not included if they had any of the following contraindications to SLIT (in accordance with [1, 5]): hypersensitivity to any of the excipients; comedication with beta-blockers; severe and/or unstable asthma (forced expiratory volume in 1 second <70% of the predicted value); severe immune deficiency or autoimmune disease; malignant diseases (e.g., cancer); oral inflammations or infections (such as oral lichen planus, oral ulcerations, or oral mycosis) [21]. However, the presence of concomitant allergies (to tree/weed pollens, house dust mites, etc.) was not a study exclusion criterion.

### 2.3. Atmospheric Grass Pollen Levels

Grass pollen counts were obtained from the German Pollen Information Service (http://www.pollenstiftung.de/). During the 2008 and 2009 grass pollen seasons, grass pollen exposure was measured with a total of 46 Burkard pollen traps operated throughout Germany by the German Pollen Information Service. Weekly atmospheric grass pollen levels (calculated as the total number of grass pollens divided by the number of pollen traps with valid data) were documented.

### 2.4. Immunotherapy

Sublingual immunotherapy was initiated about 4 months before the expected onset of the grass pollen season and was continued throughout the season (corresponding to a pre- and coseasonal regimen, in accordance with the SmPC [21]).

The five-grass pollen tablet contained freeze-dried, standardized allergen extracts of orchard (*Dactylis glomerata*), meadow (*Poa pratensis*), perennial rye (*Lolium perenne*), sweet vernal (*Anthoxanthum odoratum*), and timothy (*Phleum pratense*) grasses (Oralair, from Stallergenes SA, Antony, France). This mixture reflects the natural exposure and sensitization conditions and provides a consistent, well-balanced composition of the most common allergens [22].

The five-grass pollen tablets were to be taken in the morning. This tablet dissolves under the tongue within 1 minute. The first dose (100 index of reactivity (IR)) was administered under medical supervision and the patient was monitored for at least 30 minutes thereafter. All subsequent doses were taken at home. On the second day, the patients took two 100 IR tablets. From the third day onwards, the patients took one 300 IR tablet daily, according to the SmPC [21].

### 2.5. Outcome Measures

**Figure 1:**
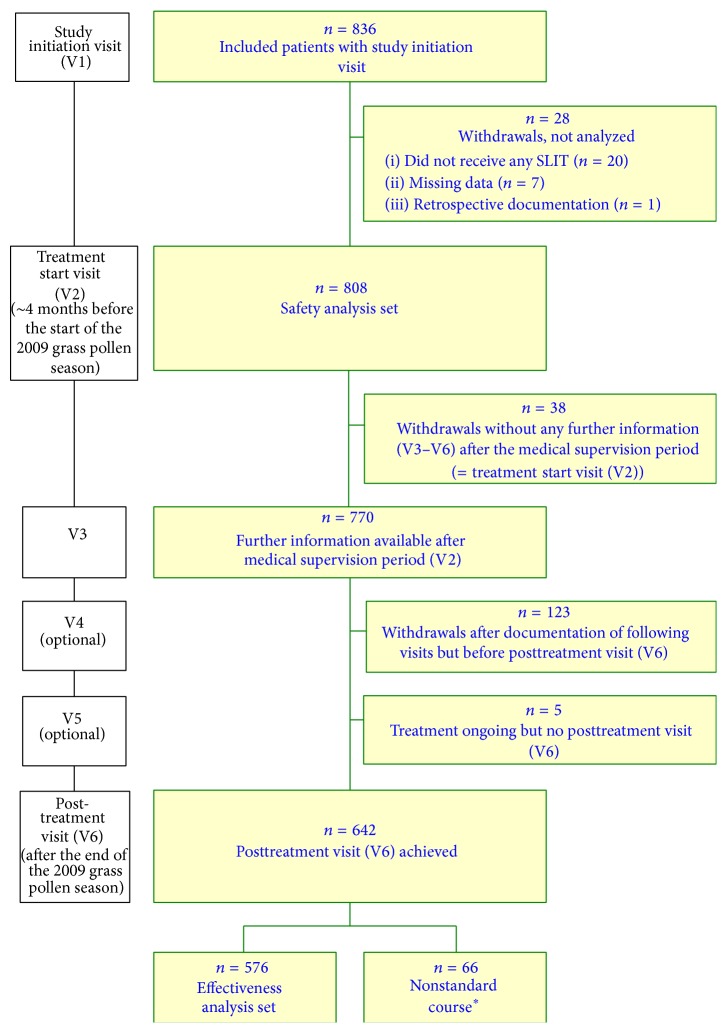
Study flow chart and patient disposition. ^∗^Nonstandard course: patients who had started the treatment during the pollen season and/or had undergone a “posttreatment” visit before the end of the pollen season.

The study flow chart is shown in Figure 1. During the study initiation visit (V1), demographic data, patient eligibility, allergy history (symptoms and other allergies), diagnostic findings, any history of AIT, concomitant diseases, concomitant medication, and symptoms and medication intake during the previous year's grass pollen season were documented. During a second visit (treatment start visit; V2), the use of any premedication (for preventing allergic reactions), any adverse drug reactions (ADRs, i.e., adverse events (AEs) possibly, probably, or certainly causally related to administration of the five-grass pollen tablet) associated with administration of the first dose, the titration mode, and the prescribed dose were recorded.

Patients subsequently attended up to three further visits (V3, V4, and V5) during the treatment period, together with a posttreatment visit (V6) in the autumn. Visits V3 to V5 were scheduled whenever a new prescription was required (depending on the prescribed package size: 30 or 90 tablets). Hence, V3, for example, was scheduled after 4 weeks of treatment or 3 months of treatment when the pack size was 30 or 90, respectively. During visits V3 to V5, data on the prescribed treatment, AEs/ADRs, and withdrawals were gathered. Lastly, the following topics were assessed at the posttreatment visit (V6): tolerability, AEs/ADRs, compliance with the prescribed treatment regimen during the titration and maintenance phases, the severity of nasal, ocular, and bronchial symptoms (on a 4-point scale: none, mild, moderate, or severe) and medication intake during the grass pollen season (using the same questions as in the study initiation visit V1), general well-being (much better, better, the same, worse, or much worse, relative to the previous season), and whether or not the patient planned to continue with AIT for the following pollen season. “Responder” patients were defined as those with less severe symptoms during the SLIT season (2009) than during the previous season (2008). AEs/ADRs were documented in terms of their nature, start time, start date, stop date, concomitant dose, seriousness, intensity, putative causality, other possible causes, treatment (if required), and outcome. Lastly, tolerability was rated on a 4-point scale (very good, good, poor, or bad) by both the investigators and the patients.

### 2.6. Study Variables and Statistical Analysis

The safety assessment was based on the incidence of AEs/ADRs. All recorded AEs/ADRs were classified according to the Medical Dictionary for Regulatory Activities (MedDRA, Version 11.1). The safety analysis set consisted of all patients having received at least one dose of the five-grass pollen tablet.

The following variables were used to assess the effectiveness of immunotherapy: the severity of nasal, ocular, and bronchial symptoms (on the four-point scale mentioned above) and the use of symptomatic medications (such as eye drops, nasal and oral antihistamines, nasal and oral corticosteroids, and inhaled corticosteroids and selective beta-2 adrenoceptor agonists). The effectiveness analysis set consisted of all patients having undergone a preseasonal examination (the study initiation visit) and a postseasonal examination (the posttreatment visit). Patients with a nonstandard course of treatment (e.g., those who had started the treatment during the pollen season and/or had undergone a “posttreatment” visit before the end of the pollen season) were not included in the effectiveness analysis set.

All variables were assessed using descriptive statistics.

## 3. Results

### 3.1. Demographic Characteristics of the Study Population

A total of 836 adult patients were initially enrolled in the study (Figure 1). Of these, 28 were excluded from the analysis due to failure to take the five-grass pollen tablet (*n* = 20), incomplete documentation (*n* = 7), or retrospective documentation (*n* = 1). Hence, a total of 808 adult patients (364 men, 444 women; mean ± standard deviation (SD) age: 34.7 ± 12.3) received at least one dose of the five-grass pollen tablet and thus constituted the safety analysis set (Table 1). Of these 808 patients, 804 (99.5%) had allergic rhinitis (with moderate-to-severe symptoms in 93.6% of cases), 625 (77.4%) had conjunctivitis (66.0% with moderate-to-severe symptoms), and 189 (23.4%) had asthma. Concomitant allergies were reported by 71.8% of patients, the most frequent concomitant allergies being caused by birch pollen (48.8%), other tree pollens (41.1%), weed pollens (28.8%), house dust mites (27.1%), and animal dander (22.8%). The majority of patients (84.0%) had taken symptomatic medications during the previous year's grass pollen season.

For 634 of the 808 patients (78.5%), the course of five-grass pollen tablets documented here was their first ever use of SLIT, whereas 146 patients (18.1%) had started SLIT with the five-grass pollen tablet sometime after they had completed another course of AIT. Lastly, the remaining 28 patients (3.5%) had switched from an ongoing course of AIT to the five-grass pollen tablet.

Of the 808 patients, 576 (71.3%) were eligible for the effectiveness evaluation (Figure 1); this somewhat low proportion was primarily due to loss to follow-up (*n* = 80, 9.9%) or early study discontinuation (*n* = 48, 5.9%) and thus the lack of a documented postseasonal assessment (*n* = 166, 20.5%). When considering the effectiveness analysis set of 576 patients, 564 (97.9%) had allergic rhinitis (with moderate-to-severe symptoms in 92.7% of cases), 497 (86.3%) had conjunctivitis, and 215 (37.3%) had asthma.

### 3.2. Atmospheric Grass Pollen Levels

**Figure 2:**
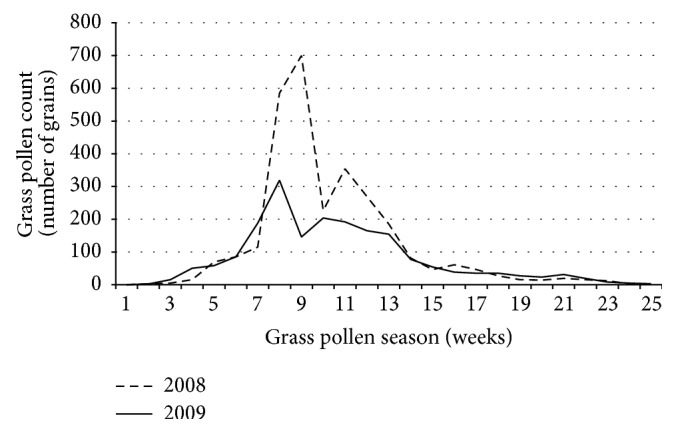
Weekly atmospheric grass pollen levels in Germany during the 2008 and 2009 grass pollen seasons (pollen data kindly provided by Professor K.-C. Bergmann, Charité, Berlin, Germany; http://www.pollenstiftung.de/).

In Germany, the overall weekly atmospheric grass pollen levels during the 2009 grass pollen season were approximately one-third lower than those recorded during the 2008 grass pollen season (Figure 2).

### 3.3. Treatment Exposure

In line with the five-grass pollen tablet's SmPC, most patients were treated with a pre- and coseasonal regimen. The preseasonal treatment was initiated between November and February for 574 of the 808 patients (71.0%), with 352 starting in December or January. In the 770 patients with further information (V3 to V6) after the first dose of the five-grass pollen tablet, the mean ± SD duration of SLIT was 191 ± 92 days and 77.4% had received treatment for more than 120 days.

In general, standard procedures were followed during the titration and maintenance phases. Very few deviations from the recommended “one tablet a day” dosage regimen were observed, with administration of half a tablet (*n* = 2), quarter of a tablet (*n* = 1), or one tablet every other day (*n* = 1). Although patients were allowed premedication (to prevent putative allergic reactions upon administration of the first five-grass pollen tablet), only 20 patients chose to use this procedure (2.5%).

### 3.4. Immunotherapy: Safety and Tolerability

More than 50% of all ADRs documented occurred upon administration of the first five-grass pollen tablet. In all, 204 of the 808 patients (25.5%) experienced at least one ADR at the time of first intake. The total number of ADRs after first intake was 419 (i.e., some patients experienced more than one ADR). The most common ADRs after first intake were gastrointestinal disorders including local reactions at the application site (*n* = 162 patients, 20.0%; including oral paresthesia (*n* = 78 patients; 9.7%) and oral pruritus (*n* = 50 patients; 6.2%)) and respiratory, thoracic, and mediastinal disorders (*n* = 69 patients; 8.5%) such as throat irritation (*n* = 52 patients; 6.4%). In terms of the time course of gastrointestinal and respiratory ADRs following the first intake of the five-grass pollen tablet, the median onset was 5 minutes and the median duration was 16 min. Further details of the time courses of these ADRs are provided in Table 2. Most of the ADRs occurring after first intake were mild to moderate in intensity, and none was serious.

Over the entire course of SLIT, 297 patients (36.8%) experienced at least one AE. There were 758 AEs in total. In 285 patients (35.3%), the investigators considered that the AEs were possibly, probably, or certainly causally related to administration of the five-grass pollen tablet (i.e., ADRs) (Table 3). The most common ADRs over the study period were gastrointestinal disorders (*n* = 222 patients; 27.5%) and respiratory disorders (*n* = 110 patients; 13.6%). Most of the local ADRs were mild-to-moderate reactions such as oral paresthesia, throat irritation, and oral pruritus.

Five patients experienced at least one serious adverse event (SAE), which were variously classified by the investigators as not related (*n* = 1), possibly related (*n* = 2), probably related (*n* = 1), and certainly related (*n* = 1) to administration of the five-grass pollen tablet. In four of these patients, the SAE was considered by the investigator to be a “medically important event,” as described hereafter (see also [19]). A 27-year-old man was diagnosed with a malignant melanoma during the study; this was classified as not related to the SLIT. After 199 days of treatment, a 20-year-old woman experienced a moderate-intensity burning sensation in her eyes. An 18-year-old man experienced severe oral pruritus on the first day of SLIT, as well as severe diarrhea, colic, and aggravation of preexisting Crohn's disease after another 15 days. A 32-year-old man experienced severe local reactions, dyspnea, and renal and chest pain after 3 days of SLIT. Furthermore, one case with the seriousness criterion “hospitalization” was reported: a 54-year-old woman was hospitalized 6 months after start of SLIT for a lumbar vertebral fracture caused by previously unsuspected stage III plasmocytoma. Allergen immunotherapy is not known to induce malignant diseases, and so the diagnosis of a stage III plasmocytoma 6 months after starting SLIT was considered unlikely to have a chronologic, causal relationship to SLIT.

No severe or life-threatening (anaphylactic) systemic reactions were recorded during the study.

In total, 75 patients (9.8%, which includes the 5 patients with SAEs) terminated their treatment prematurely due to AEs/ADRs.

Remedial drug therapy for ADRs (in a total of 49 patients (6.1%)) consisted predominantly of symptomatic medications (mainly antihistamines). The tolerability of the five-grass pollen tablet was rated as “good” to “very good” by 90.0% of the investigators and 86.7% of the patients.

### 3.5. Immunotherapy: Effectiveness

A total of 576 patients with a documented postseasonal assessment (V6) retrospectively rated their symptom severity for the grass pollen season and thus were eligible for the effectiveness evaluation (as they had done at the study initiation visit for the previous pollen season).

84.9% of the patients with nasal symptoms were classified as responders (i.e., with less severe symptoms or no symptoms, relative to the previous season), and about a quarter of these were symptom-free. The severity of nasal symptoms remained unchanged in 13.1% of patients and had worsened in 2.1%. For patients with eye symptoms, the response rate was 76.8% (two-fifths of whom were symptom-free). The response rates for concomitant bronchial and skin symptoms (73.3% and 71.8%, resp.) were similar to those for nasal and eye symptoms.

The reduction in the severity of nasal, ocular, and bronchial symptoms during the first season on SLIT (relative to the preceding grass pollen season) suggested that this treatment was associated with clinical benefit (Table 4). In particular, very few of the patients (between 0.7% and 5.4%) experienced severe symptoms during the first season of SLIT. In contrast, the number of patients reporting the absence of nasal symptoms increased substantially from 6 (1.1%) in the preceding season to 119 (20.9%) during the first season on SLIT.

Data on the use of symptomatic medications in the preceding pollen season and the first season on SLIT were available for a total of 567 patients. During the grass pollen season preceding the initiation of SLIT, 483 patients (85.2%) had used some type of symptomatic medication and 84 patients (14.8%) had not used any. During the course of SLIT, the number of patients not using any such medication increased to 261 (46.0%). Overall, the proportion of patients needing to take symptomatic medications decreased by 36.6% during SLIT. When considering all the individual classes of symptomatic medication, more than half of the patients no longer needed to take these drugs during the SLIT season (Table 5).

When questioned, 86.7% of the patients stated that they had a higher or much higher level of well-being during the SLIT season than during the preceding season. This opinion was shared by 87.9% of the investigators.

## 4. Discussion

Current guidelines (such as the PRACTALL consensus report from the European Academy of Allergy and Clinical Immunology and the American Academy of Allergy and Clinical Immunology) emphasize the value of postmarketing, noninterventional studies in assessing the “true” effectiveness of AIT in routine medical practice [2]. We performed such a “real-life,” open, prospective, noninterventional study over a single pollen season and assessed pre-/coseasonal SLIT with a five-grass pollen tablet in 808 adult patients with grass pollen allergy. The large number of investigating centers (354) helped to avoid bias and ensured that our analysis was representative of real-life conditions, as proposed by Pfaar et al. [17].

The majority of our patients suffered from moderate-to-severe symptoms of allergic rhinitis and more than three-quarters also suffered from conjunctivitis.

Although effectiveness was assessed (see below), the study's primary objective was to assess the safety and tolerability of pre- and coseasonal treatment with five-grass pollen tablets. About a quarter of patients experienced at least one ADR on the first day of treatment. However, over the course of treatment as a whole, fewer than 40% of patients experienced one or more ADRs. The observed ADRs were mainly gastrointestinal (e.g., oral paresthesia and pruritus) and respiratory (e.g., throat irritation) in nature. The majority of the ADRs were mild to moderate in intensity. Taken as a whole, these data clearly indicate that routine SLIT with five-grass pollen tablets had a good safety profile in a cohort of more than 800 patients with grass pollen allergy. Although SADRs were documented in four patients, no severe (anaphylactic) systemic reactions were observed.

When compared with RCTs of the five-grass pollen tablet [8], the proportion of patients reporting AEs in the present noninterventional study was lower and the proportion of patients prematurely terminating the study due to AEs was higher (5.2% in an RCT [8] versus 9.8% in the present study). This finding may be due to differences in methodological settings when comparing noninterventional, observational studies with DBPC trials; the latter are based on carefully controlled clinical protocols with smaller numbers of enrolled and analyzed participants [17].

According to the German SmPC for the five-grass pollen tablet [21], the first dose must be administered under medical supervision. Given that nearly 50% of the ADRs in the present study (none of which was serious) occurred within 30 minutes of the first intake, this recommendation appears to be justified. Even though none of the ADRs following first intake was serious and the SLIT's overall safety profile was excellent, four serious ADRs were nevertheless recorded at some point during the study. Since patient safety is the prime concern, initial administration under medical supervision is worthwhile and also gives the physician an opportunity to discuss the management of potential AEs with the patient.

Almost 10% of the patients studied here (including the five with SAEs) terminated SLIT prematurely due to AEs/ADRs. This underlines the importance of thorough, continuous communication between physicians and patients before treatment start, in order to provide comprehensive information on the possible occurrence of SLIT-related ADRs and ensure that appropriate action is taken if an ADR occurs. This should increase adherence to treatment.

Secondly, the present study was designed to analyze clinical effects of SLIT in the first season of pre- and coseasonal treatment (relative to the preceding grass pollen season). More than 70% of the patients considered that their nasal, ocular, and bronchial symptoms were less severe during the SLIT season than during the preceding season. For all the individual classes of symptomatic medication used during the grass pollen season preceding SLIT, more than 50% of the corresponding patients discontinued their use of these drugs during the SLIT season. This decrease in symptomatic medication intake evidences a substantially lower disease burden.

However, our ability to interpret clinical effects in this open, noninterventional, noncontrolled study was somewhat limited by (i) the absence of a placebo group and (ii) the fact that atmospheric grass pollen levels in Germany were approximately one-third lower during the SLIT season (2009) than during the preceding grass pollen season (2008). Therefore, the degree of symptom relief and the decrease in symptomatic medication use observed during the SLIT season might also have been due to (i) some degree of placebo effect and (ii) lower levels of exposure to grass pollens. Indeed, the European Medicines Agency's current guideline emphasizes that “the unpredictability and variability of allergen exposure especially to pollen allergens” remains a problem for clinical trials in AIT in general [23] and may limit the value of information gained from noninterventional, open studies. Lastly, our ability to draw conclusions about clinical effectiveness was limited by the fact that 28.7% of the study participants were not eligible for the effectiveness evaluation (due primarily to loss to follow-up and early study discontinuation).

## 5. Conclusions

Under real-life conditions, SLIT with a five-grass pollen tablet was well tolerated in a population of more than 800 adult patients with grass pollen allergy, thus confirming the results of the many preceding RCTs. Postmarketing, noninterventional studies are of great value for assessing the safety of AIT products in routine medical practice, as recommended in recent guidelines.

Our data indicated that the five-grass pollen tablet was associated with both good tolerability and symptom relief (including considerably less symptomatic medication use). Despite the difference in atmospheric grass pollen levels between the season preceding SLIT and the first season of SLIT (which made it difficult to draw definitive conclusions on effectiveness), the stated degree of symptom relief suggests that SLIT was indeed associated with clinical benefits.

## Figures and Tables

**Table 1:** Patient characteristics.

Parameter	Safety analysis set
	*n* = 808
Age	
Mean ± SD [years]	34.7 ± 12.3
Median [years]	33.0
Range [years]	18–82
Sex	
Male [*n* (%)]	364 (45.0)
Female [*n* (%)]	444 (55.0)
Symptoms	
Allergic rhinitis [*n* (%)]	804 (99.5)
Severity in the grass pollen season 2008 [*n* (%)]	
Mild	41 (5.1)
Moderate	323 (40.0)
Severe	433 (53.6)
Allergic conjunctivitis [*n* (%)]	625 (77.4)
Severity in the grass pollen season 2008 [*n* (%)]	
Mild	167 (20.7)
Moderate	301 (37.3)
Severe	232 (28.7)
Allergic asthma [*n* (%)]	189 (23.4)
Allergy history	
Mean ± SD duration since first occurrence [years]	
Allergic rhinitis	10.2 ± 9.0
Allergic conjunctivitis	10.7 ± 9.2
Allergic asthma	9.2 ± 9.7
History of immunotherapy [*n* (%)]	
No (first time immunotherapy)	634 (78.5)
Yes, completed	146 (18.1)
Yes, switch from an ongoing immunotherapy	28 (3.5)
Concomitant allergies	
Yes [*n* (%)]	580 (71.8)
Concomitant allergies to [*n* (%)]	
Birch pollen	394 (48.8)
Other tree pollens	332 (41.1)
Weed pollens	233 (28.8)
House dust mites	219 (27.1)
Animal dander	184 (22.8)
Moulds	71 (8.8)
Symptomatic medication	
Symptomatic medication intake during the grass pollen season 2008 [*n* (%)]	679 (84.0)

*n*: number of patients; %: percentage of patients.

**Table 2:** Time course of adverse drug reactions (ADRs) after the first intake of the five-grass pollen tablet.

MedDRA system organ class and low-level term (LLT)	Time lag between first intake and onset median (range) [minutes]	Duration median (range) [minutes]
Gastrointestinal disorders	5 (0–570)	16 (1 min–63.5 hours)
Tingling mouth	5 (0–20)	14 (2–180)
Oral pruritus	5 (0–60)	15 (2–180)
Respiratory, thoracic, and mediastinal disorders	5 (0–645)	15.5 (2–360)
Throat irritation	5 (0–645)	10 (2–75)

MedDRA: Medical Dictionary for Regulatory Activities, Version 11.1.

**Table 3:** Adverse events (in general and those related to treatment, i.e., adverse drug reactions) having occurred in at least 1% of the 808 patients during the first year of SLIT.

MedDRA system organ class and preferred term (PT)	Number of patients (%)	Number of events
AEs (total)	297 (36.8)	758
Treatment-related AEs (=ADRs) (total)	285 (35.3)	720
Ear and labyrinth disorders	17 (2.1)	18
Ear pruritus	15 (1.9)	16
Eye disorders	14 (1.7)	19
Eye pruritus	8 (1.0)	9
Gastrointestinal disorders	222 (27.5)	411
Paresthesia oral	88 (10.9)	118
Oral pruritus	56 (6.9)	74
Edema mouth	43 (5.3)	52
Oral discomfort	20 (2.5)	24
Nausea	16 (2.0)	18
Lip swelling	12 (1.5)	13
Glossodynia	11 (1.4)	15
Tongue edema	11 (1.4)	11
Hypoesthesia oral	10 (1.2)	10
Dyspepsia	9 (1.1)	11
Dysphagia	9 (1.1)	11
Nervous system disorders	26 (3.2)	31
Paresthesia	9 (1.1)	12
Respiratory, thoracic, and mediastinal disorders	110 (13.6)	163
Throat irritation	62 (7.7)	80
Pharyngeal edema	14 (1.7)	16
Dyspnea	10 (1.2)	12
Sneezing	9 (1.1)	9
Cough	8 (1.0)	8
Skin and subcutaneous tissue disorders	25 (3.1)	35
Pruritus	15 (1.9)	17

MedDRA: Medical Dictionary for Regulatory Activities, Version 11.1.

%: percentage of patients; AE: adverse event; ADR: adverse drug reaction.

**Table 4:** Nasal, ocular, and bronchial symptoms of grass pollen allergy during the 2008 grass pollen season (the reference season) and the 2009 season (the first year of SLIT).

Symptoms	Severity	Reference season	Immunotherapy season
		*n* (%)	*n* (%)
Nasal symptoms	All patients with data	570 (100)	570 (100)
	No symptoms	6 (1.1)	119 (20.9)
	Mild	30 (5.3)	300 (52.6)
	Moderate	217 (38.1)	120 (21.1)
	Severe	317 (55.6)	31 (5.4)

Ocular symptoms	All patients with data	566 (100)	566 (100)
	No symptoms	69 (12.2)	228 (40.3)
	Mild	113 (20.0)	241 (42.6)
	Moderate	214 (37.8)	75 (13.3)
	Severe	170 (30.0)	22 (3.9)

Bronchial symptoms	All patients with data	546 (100)	546 (100)
	No symptoms	331 (60.6)	442 (81.0)
	Mild	108 (19.8)	76 (13.9)
	Moderate	82 (15.0)	24 (4.4)
	Severe	25 (4.6)	4 (0.7)

*n*: number of patients; %: percentage of patients.

**Table 5:** Number of patients using symptomatic medications during the 2008 grass pollen season (the reference season) and discontinuing these symptomatic medications during the 2009 season (the first year of SLIT).

Type of medication	Symptomatic medication use during the pollen season	
	Medication used in 2008	Medication used in 2008 and *discontinued* during SLIT in 2009
	*n*	*n* (%)
Eye drops	209	149 (71.3)
Nasal antihistamines	150	129 (86.0)
Oral antihistamines	388	222 (57.2)
Nasal corticosteroids	135	95 (70.4)
Oral corticosteroids	28	26 (92.9)
Inhaled corticosteroids	64	39 (60.9)
Inhaled beta-2 adrenoceptor agonists	69	37 (53.6)

*n*: number of patients; %: percentage of patients.

## Abbreviations

ADR: Adverse drug reaction
AE: Adverse event
AIT: Allergen immunotherapy
ARC: Allergic rhinoconjunctivitis
DBPC: Double-blind placebo-controlled
IR: Index of reactivity
RCT: Randomized controlled trial
SAE: Serious adverse event
SCIT: Subcutaneous immunotherapy
SLIT: Sublingual immunotherapy
SmPC: Summary of product characteristics.
